# Molecular identification of protein kinase C beta in Alzheimer's disease

**DOI:** 10.18632/aging.103994

**Published:** 2020-11-07

**Authors:** Zhike Zhou, Fenqin Chen, Shanshan Zhong, Yi Zhou, Rongwei Zhang, Kexin Kang, Xiaoqian Zhang, Ying Xu, Mei Zhao, Chuansheng Zhao

**Affiliations:** 1 Department of Geriatrics, The First Affiliated Hospital, China Medical University, Shenyang, Liaoning, PR China; 2 Department of Neurology, The First Affiliated Hospital, China Medical University, Shenyang, Liaoning, PR China; 3 Computational Systems Biology Lab, Department of Biochemistry and Molecular Biology and Institute of Bioinformatics, The University of Georgia, Athens, GA 30602, USA; 4 Cancer Systems Biology Center, The China-Japan Union Hospital, Jilin University, Changchun, PR China; 5 Department of Cardiology, The Shengjing Affiliated Hospital, China Medical University, Shenyang, Liaoning, PR China

**Keywords:** PRKCB, Alzheimer's disease, gene expression, network

## Abstract

The purpose of this study was to investigate the potential roles of protein kinase C beta (PRKCB) in the pathogenesis of Alzheimer’s disease (AD). We identified 2,254 differentially expressed genes from 19,245 background genes in AD versus control as well as PRKCB-low versus high group. Five co-expression modules were constructed by weight gene correlation network analysis. Among them, the 1,222 genes of the turquoise module had the strongest relation to AD and those with low PRKCB expression, which were enriched in apoptosis, axon guidance, gap junction, Fc gamma receptor (FcγR)-mediated phagocytosis, mitogen-activated protein kinase (MAPK) and vascular endothelial growth factor (VEGF) signaling pathways. The intersection pathways of PRKCB in AD were determined, including gap junction, FcγR-mediated phagocytosis, MAPK and VEGF signaling pathways. Based on the performance evaluation of the area under the curve of 75.3%, PRKCB could accurately predict the onset of AD. Therefore, low expressions of PRKCB was a potential causative factor of AD, which might be involved in gap junction, FcγR-mediated phagocytosis, MAPK and VEGF signaling pathways.

## INTRODUCTION

Alzheimer’s disease (AD), referring to a chronic, acquired and progressive impairment of cognition, is characterized pathologically by the extracellular accumulation of amyloid beta (Aβ) and intracellular inclusions of phosphorylated tau protein [1]. An estimated population of at least 20 million worldwide suffer from this clinical entity, with its attendant enormous human and financial burden on society and families [2]. Since the course of AD cannot be postponed or reversed by the available clinical interventions, many efforts are directed towards symptomatic relief similar to palliative care. Practically, there is a growing concern regarding preventive medicine related to risk factors for AD, including lifestyles, environment, comorbidities, and genetic predisposition [3–5].

Mutations in genes such as presenilin 1, presenilin 2, amyloid precursor protein and apolipoprotein E have been confirmed to increase the risk of AD [4]. Protein kinase C beta (PRKCB), an enzyme in the serine-threonine kinase family, has been implicated in the conversion of extracellular signals to biological responses with its functional changes possibly contributing to the development of AD [6]. Both in vitro and in vivo experiments demonstrated that early reduction of protein kinase C was an essential step and a prognostic feature of excitotoxic neuronal death [7]. Nevertheless, the mechanisms of PRKCB underlying the progress of AD were not well understood. We performed an integrated analysis of PRKCB based on gene expression data in AD and functional annotations, aiming to elucidate the potential roles of PRKCB in the pathogenesis of AD.

## RESULTS

### Identification of differentially expressed genes

**Figure 1:**
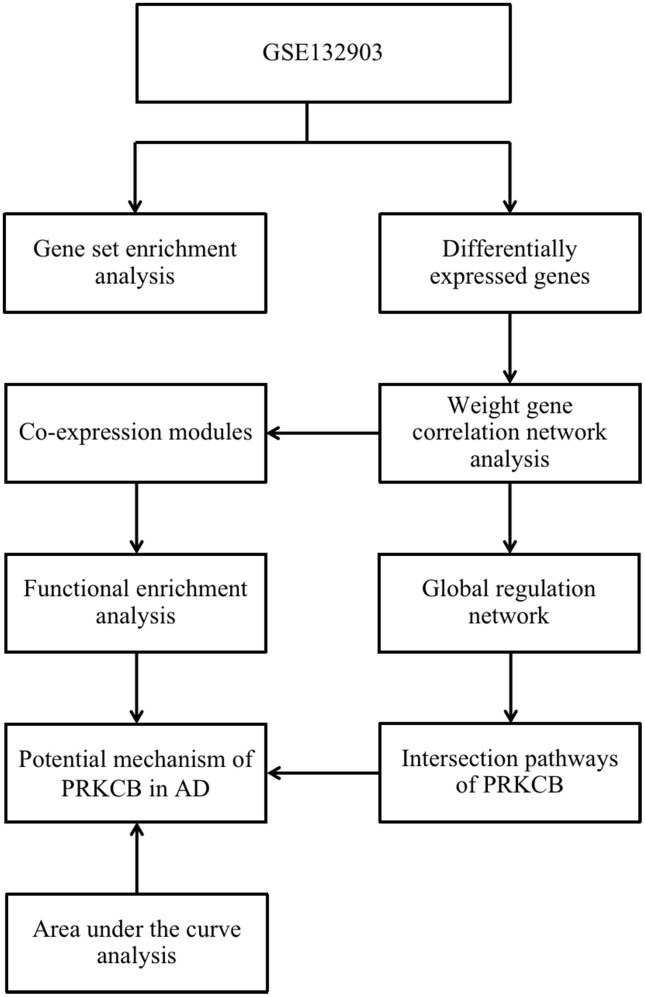
**The roadmap of the present study.** AD: Alzheimer’s disease.

**Figure 2:**
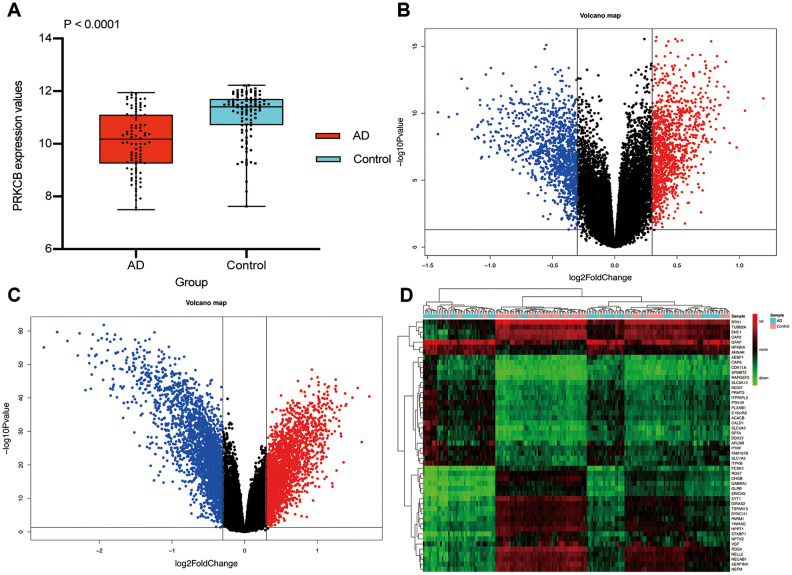
**Differential expression gene analysis.** The expression of PRKCB in AD and non-dementia controls (**A**) Volcano plot of the AD / control (**B**) and PRKCB-low / high group (**C**) blue, black and red indicate down-regulated, non-significant and up-regulated DEGs, respectively. The heatmap of the top 25 down-regulated and up-regulated DEGs (**D**) AD: Alzheimer’s disease, DEGs: differential expression genes.

The conceptual level roadmap is shown in Figure 1. According to the gene data of all samples, the mean expressions of PRKCB in 97 AD patients (10.08 ± 1.16) were significantly lower than those in 98 non-dementia controls (11.07 ± 0.95; P < 0.001) (Figure 2A). Totally 19,245 background genes were selected in our differential expression analyses after having removed the unannotated and duplicated genes. Among them, 2,365 DEGs with 1,205 down-regulated and 1,160 up-regulated were established in AD patients compared to controls (Figure 2B); whereas 4,679 DEGs with 2,356 down-regulated and 2,323 up-regulated were identified in subjects with low versus high expression of PRKCB (Figure 2C). There were 2,254 overlapping DEGs in AD / control and PRKCB-low / high groups. The top 25 down-regulated and up-regulated overlapping DEGs associated with AD and low PRKCB expression were represented as a heatmap (Figure 2D).

### Co-expression modules and functional enrichment analysis

**Figure 3:**
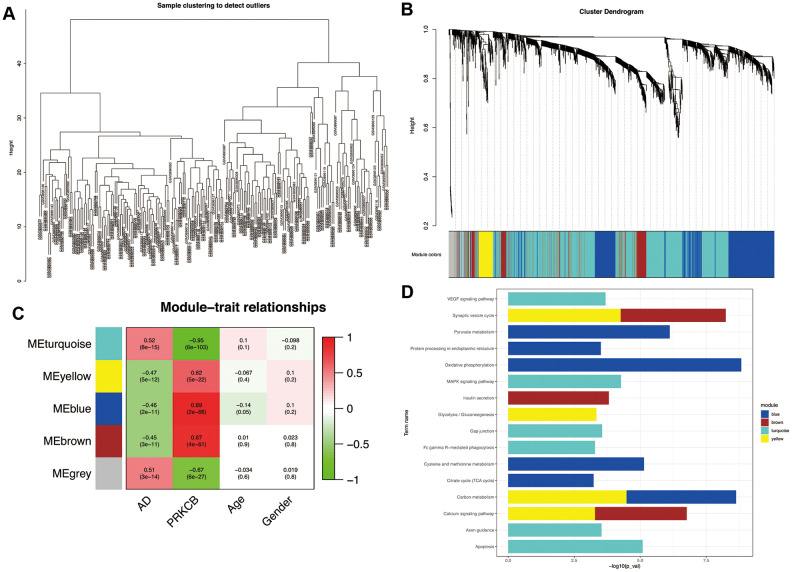
**Weighted correlation network analysis.** All the samples were included in the clusters (**A**) Cluster dendrogram of five modules with different colors (**B**) grey represents non-clustering genes. The heatmap of module-trait relationships (**C**) red indicates positive correlation and green represents negative correlation. Enrichment analysis of KEGG pathways in co-expression modules (**D**) AD: Alzheimer’s disease, KEGG: Kyoto Encyclopedia of Genes and Genomes.

The results of our sample clustering showed that all the samples passed the predefined cutoff and were included in some clusters (Figure 3A). Five co-expression modules with different colors were predicted by WGCNA based on the expression data of DEGs associated with PRKCB and AD (Figure 3B). The modules colored in blue, brown, turquoise and yellow consist of 627, 226, 1,222 and 101 DEGs, respectively. As shown in Figure 3C, the turquoise module positively correlated with AD (correlation coefficient = 0.52, P = 8e-15) and negatively correlated with PRKCB expression (correlation coefficient = -0.95, P = 6e-103), whilst the blue, brown and yellow modules all had a negative correlation with AD (blue: correlation coefficient = -0.46, P = 2e-11; brown: correlation coefficient = -0.46, P = 2e-11; yellow: correlation coefficient = -0.47, P = 5e-11) and positive correlation with PRKCB expression (blue: correlation coefficient = 0.89, P = 2e-66; brown: correlation coefficient = 0.87, P = 4e-61; yellow: correlation coefficient = 0.62, P = 5e-22). Functional enrichment analyses (Figure 3D) revealed that the DEGs of the blue module were involved in KEGG pathways of oxidative phosphorylation, pyruvate metabolism and protein processing in endoplasmic reticulum; the brown and yellow modules DEGs were enriched in synaptic vesicle cycle and calcium signaling pathways; the DEGs of the turquoise module participated in apoptosis, axon guidance, gap junction, Fc gamma receptor (FcγR)-mediated phagocytosis, mitogen-activated protein kinase (MAPK) and vascular endothelial growth factor (VEGF) signaling pathways.

### Global regulation network and AUC analysis of PRKCB

**Figure 4:**
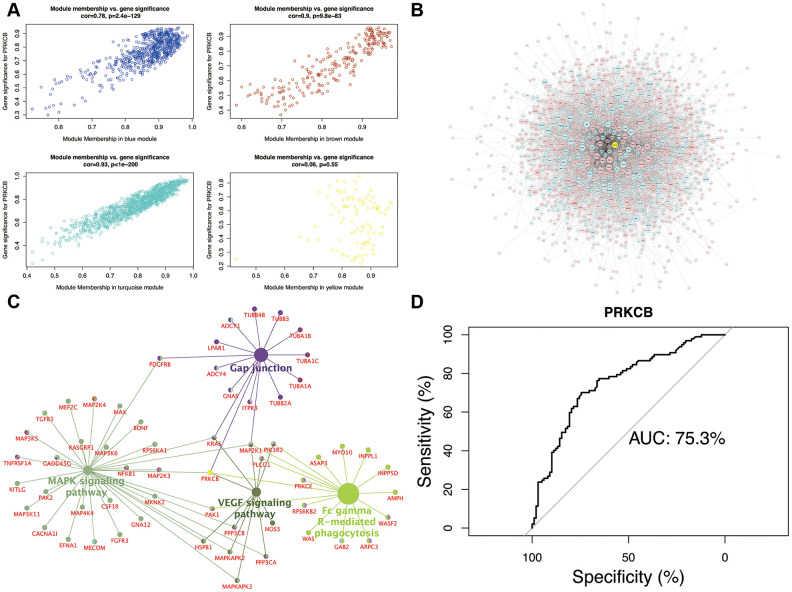
**Module-pathway regulatory network and AUC analysis.** Scatterplot of module membership vs. gene significance (**A**) Global regulatory network of turquoise module (**B**) node size represents the degree of gene connectivity; yellow and blue indicate low expression of gene, whilst red represents high expression. The intersection pathways of PRKCB (**C**) yellow indicates the low PRKCB expression. Performance evaluation of AUC analysis (**D**) AUC: area under the curve.

In the scatter plot of the relationships between GS and MM (Figure 4A), the intramodular connectivity within the turquoise module most closely correlated with the genetic phenotypes (correlation coefficient = 0.93, P = 1e-200). Low expression of PRKCB interacting with DEGs in the turquoise module was exhibited in the global regulation network (Figure 4B). The intersection pathways of PRKCB, such as gap junction, FcγR-mediated phagocytosis, MAPK and VEGF signaling pathways, were identified, and all the genes enriching these pathways were shown in Figure 4C. The performance evaluation of PRKCB in predicting AD was measured using the AUC analysis (AUC = 75.3%) (Figure 4D).

### GESA verification in biological processes

**Figure 5:**
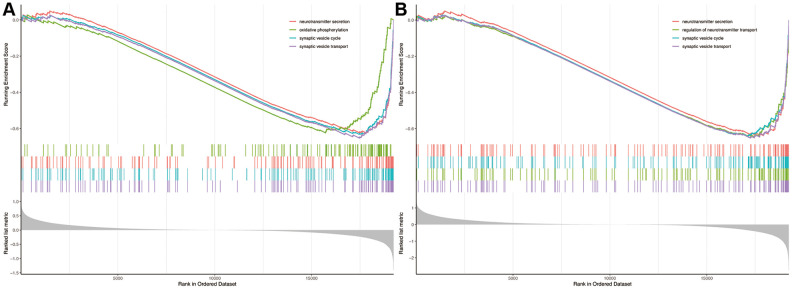
**Gene set enrichment analysis.** Biological processes enriched in AD (**A**) and low expression of PRKCB (**B**). AD: Alzheimer’s disease.

The gene set enrichment analysis showed that biological processes of neurotransmitter secretion, oxidative phosphorylation, synaptic vesicle cycle and synaptic vesicle transport were significantly enriched in AD patients compared to non-dementia controls (Figure 5A). Likewise, biological processes of neurotransmitter secretion, regulation of neurotransmitter transport, synaptic vesicle cycle and synaptic vesicle transport were significantly enriched in PRKCB-low expression versus high expression group (Figure 5B).

## DISCUSSION

In the present study, a total of 195 subjects involving 19,245 genes were analyzed to investigate the relationship between PRKCB and the incidence of AD. The results of GSEA revealed that DEGs in both AD/ control and PRKCB-low/ high cohorts enriched biological processes of synaptic vesicle cycle, synaptic vesicle transport and neurotransmitter secretion. With the exception of a steady and unanimous pathology in AD, abnormalities in synapse and neurotransmitter were the primary configurational correlations with cognitive severity [8–10]. Of particular note was that these processes were possibly linked to AD as well as the low expression of PRKCB. Thereafter, the regulatory network and co-expression modules of DEGs associated with PRKCB were constructed for further exploration, which could deepen the knowledge into the genome-scale pathogenesis of PRKCB in AD.

In line with the GSEA results, functional enrichment analysis showed that DEGs in the brown and yellow modules participated in KEGG pathway of synaptic vesicle cycle, supporting its fundamental process for AD development. More specifically, as a prime site for the production and toxicity of Aβ polymers, synaptic vesicle cycle held a central role in the presynaptic terminal pathology of AD [11]. The turquoise module exhibited the greatest negative correlation with AD and PRKCB in our results, hence supporting the involvement of DEGs in apoptosis, axon guidance, gap junction, FcγR-mediated phagocytosis, MAPK and VEGF signaling pathways. Among them, neuronal death of hippocampus in AD was the result of an apoptotic mechanism [12] and MAPK signaling pathway engaged irreversible cellular decisions of apoptosis [13]. PRKCB, a highly expressed member of protein kinase C, was activated by the second messengers Ca^2+^ and diacylglycerol to mediate the proliferation of cells [14]. Its localization in mitochondria was found to increase the hypoxic stress and vascular dysfunction, and trigger the MAPK signaling pathway of phosphorylation linked to AD progression [15, 16]. In vitro model of PRKCB deficient cells, the development of germinal centers was impeded by the impairment of antigen polarization and presentation, a remarkable finding given that PRKCB was largely considered as the dominator of cell-fate tendency [17].

Apart from the MAPK signaling pathway, the analysis of intersection pathways revealed that PRKCB was jointly involved in gap junction, FcγR-mediated phagocytosis and VEGF signaling pathway. Less is known about the association of PRKCB with gap junction and FcγR-mediated phagocytosis. In AD patients and murine models of familial AD, gap junction communication in astrocytes contacting amyloid plaques was attributed to the expression of connexins, which were reversed by protein kinase C inhibitors [18, 19]. However, this involvement in AD pathology was only beginning to be appreciated. Phagocytosis is a complicated process involving the synergistic effects of signal-transduction cascades, resulting in ingestion, subsequent phagolysosome fusion, and oxidative activation [20]. FcγR and complement receptor 3 are two well-studied types of phagocytic receptors, both of which recruit Arp2/3 complex-mediated actin polymerization and particle internalization to phagocytic activity of microglia [21, 22]. The inflammatory response in AD was initiated by the complement system [23], which up-regulated microglial phagocytosis through the activation and migration of immune cells [24]. There was evidence that the complement activation and its products such as the membrane attack complex (C5b-9) were exposed to amyloid plaques in AD brains [25, 26]. In non-radioactive in situ hybridization, transcripts encoding complement C1q and C3 participated in the neuronal degeneration in the frontal cortex of AD [27]. Additionally, as a downstream adaptor of complement receptor 3, TYRO protein tyrosine kinase-binding protein was reported to be responsible for the clearance of Aβ and apoptotic neurons, which supported the involvement of complement receptor in the pathogenesis of AD [28]. PRKCB related to superoxide generation and activation of FcγR-mediated phagocytosis in microglia, had also raised concerns on the context of potential treatment for AD [29, 30]. A recent study in mouse models of AD showed that FcγR binding to Aβ peptides facilitated the oxidative phosphorylation of tau protein [31], which was consistent with our enrichment analysis of the blue module. Intriguingly, this effect was alleviated by FcγR knockout in neurons or antagonizing its connection with Aβ [31]. Besides, several studies reported that VEGF was not only involved in providing pro-survival signals, but also drove extracellular calcium influx and expression of the PKCB gene [32, 33]. Increased intracellular calcium further expedited the activation of protein kinase C, leading to the activator protein-1 overexpression, which in turn promoted the up-regulation of VEGF [34]. In case of the subnormal VEGF, vascular insufficiency ensued with aggravated cerebral hypoperfusion and impaired clearance of Aβ [35, 36]. The resultant accumulation of Aβ prevented the bond of VEGF to its receptors against the angiogenic activity, and thus to neuronal dysfunction and loss [37].

On the basis of the scatter plot between MM and GS, the turquoise module with the highest degree of correlation coefficient validated the strongest interactions of its DEGs with the PRKCB expression. Subsequently, DEGs of the turquoise module were displayed in the global regulatory network to identify the intersection pathways of PRKCB, which supported the pleiotropic roles of PRKCB in AD pathophysiology responsible for gap junction [18], FcγR-mediated phagocytosis [31], MAPK and VEGF signaling pathways [16, 38]. Due to the low expression of PRKCB, the vulnerability of relevant pathways might be strikingly apparent, resulting in the occurrence of AD under the comprehensive pathogenic effects. Furthermore, the diagnostic performance of PRKCB showed an AUC value of 75.3%, which implied that PRKCB might be served as a predictive factor for the incidence of AD. There was a lack of gene expression confirmation using reverse transcription-polymerase chain reaction or Western blot or a combination of both. However, previous study using quantitative polymerase chain reaction revealed that Enzastaurin, a selective PRKCB inhibitor, reduced the overnight retention and presented worse performance on the latter testing day, indicating that the decreased PRKCB expression might contribute to impaired hippocampal learning and memory function [39]. Future experiments in cells or in vivo were needed to validate the presented mechanisms of PRKCB in AD, particularly with regard to gap junction and FcγR-mediated phagocytosis.

## CONCLUSIONS

In aggregate, the results emerging from the current study provided support that bioinformatic analysis was a promising approach to elucidate complicated pathways underlying the AD onset. Low expression of PRKCB had important implications in the pathogenesis of AD, possibly associated with gap junction, FcγR-mediated phagocytosis, MAPK and VEGF signaling pathways.

## MATERIALS AND METHODS

### Data resources

RNA expression data of middle temporal gyrus and clinical traits between 97 AD patients and 98 non-dementia controls were obtained from the GSE132903 dataset of Gene Expression Omnibus (GEO, https://www.ncbi.nlm.nih.gov/geo/) database [40]. According to the clinical data recorded in a previous study [41], the two groups were age-and sex-matched. The mean age was 85.02 ± 6.75 years (range: 70-98 years) for AD and 84.98 ± 6.90 years (range: 70-102) for non-dementia. Although individual data on medication and dementia severity were unavailable, we found that mean Mini-mental State Examination (MMSE) of the case group was 12.14 ± 9.21 (range: 0-28), indicating moderate dementia, whilst that of non-dementia controls was 28.12 ± 1.76 (range: 24-30). This dataset was produced on the platform of GPL10558 using the Illumina HumanHT-12 V4.0 arrays. A gene that corresponded to multiple probes retained the one with the highest value of expression. In the limma package of R software version 3.6.2, normalizeBetweenArrays function was applied for normalization processing of the gene expression data [42].

### Gene set enrichment analysis (GSEA)

The biological process of Gene Ontology terms potentially linked to AD and low PRKCB expression were filtrated through the analysis of GSEA [43, 44]. The permutation of 1000 times was performed using default weight statistic with the normalized P < 0.05 as the threshold for significant enrichment. The packages of ClusterProfler, ggplot2, enrichplot and GSEABase were utilized to accomplish the visualization of enrichment data in GSEA analysis.

### Identification of differentially expressed genes (DEGs)

The included samples were dichotomized into PRKCB-low and high cohorts according to the average expression value of PRKCB as the cut-off point. The DEGs of AD / control and PRKCB-low / high groups were respectively established by adapting lmFit and eBayes functions of limma packages. A false discovery rate (FDR)-adjusted P < 0.05 and logFC (fold change) > 0.3 were considered to be statistically significant in the differential analysis of gene expression [42, 45].

### Weight gene correlation network analysis (WGCNA)

An unbiased gene co-expression analysis on the overlapping DEGs between AD / control and PRKCB-low / high cohorts was conducted by WGCNA. The unique advantage of WGCNA was that it transformed complicated data of gene expression into modules of co-expressed genes, providing in-depth understanding of signal networks that were possibly responsible for phenotypic traits of interest [46]. Not only could it contribute to the process of comparing DEGs, but also help in the identification of gene co-expression modules specific to the disease [47]. The clustering dendrogram was plotted to exclude the sample outliers using the hclust function. During the process of module construction, the pickSoftThreshold function was utilized to screen the soft threshold, and an appropriate power of 15 was determined to maintain the degree of independence higher than 0.8. Co-expression modules with different color labels were constructed using the WGCNA package [48]. The minimum numbers of genes in each module were set to 30 for the reliability of the results. Functional enrichment analysis was carried out to filtrate genes that were enriched in Kyoto Encyclopedia of Genes and Genomes (KEGG) pathways using the clusterProfiler package; the enrichment of FDR < 0.05 was statistically significant.

### Construction of global regulatory network and intersection pathways of PRKCB

The verboseScatterplot function was used to plot the scatter diagram on the relationship between module membership (MM) and gene significance (GS), which represented intramodular connectivity and genetic phenotypes, respectively [49]. Based on the STRING database (Search Tool for the Retrieval of Interacting Genes, https://www.string-db.org/) [50], the module with the highest degree of correlation were determined to construct the global regulatory network. Then, cytoscape software was adapted to visualize the global regulatory network and the intersection pathways of PRKCB [51].

### Analysis of area under the curve (AUC)

The performance of PRKCB in differentiating AD from non-dementia was estimated by using pROC function. In general, a random selection was indicated by an AUC value of 50%, and a complete prediction was represented by 100%. All P values were bilateral, and those less than 0.05 were considered as statistically significant.
